# Twin-Schnorr: A Security Upgrade for the Schnorr Identity-Based Identification Scheme

**DOI:** 10.1155/2015/237514

**Published:** 2015-01-27

**Authors:** Ji-Jian Chin, Syh-Yuan Tan, Swee-Huay Heng, Raphael Chung-Wei Phan

**Affiliations:** ^1^Faculty of Engineering, Multimedia University, 63000 Cyberjaya, Selangor, Malaysia; ^2^Faculty of Information Science and Technology, Multimedia University, 75450 Melaka, Malaysia

## Abstract

Most identity-based identification (IBI) schemes proposed in recent literature are built using pairing operations. This decreases efficiency due to the high operation costs of pairings. Furthermore, most of these IBI schemes are proven to be secure against impersonation under active and concurrent attacks using interactive assumptions such as the one-more RSA inversion assumption or the one-more discrete logarithm assumption, translating to weaker security guarantees due to the interactive nature of these assumptions. The Schnorr-IBI scheme was first proposed through the Kurosawa-Heng transformation from the Schnorr signature. It remains one of the fastest yet most secure IBI schemes under impersonation against passive attacks due to its pairing-free design. However, when required to be secure against impersonators under active and concurrent attacks, it deteriorates greatly in terms of efficiency due to the protocol having to be repeated multiple times. In this paper, we upgrade the Schnorr-IBI scheme to be secure against impersonation under active and concurrent attacks using only the classical discrete logarithm assumption. This translates to a higher degree of security guarantee with only some minor increments in operational costs. Furthermore, because the scheme operates without pairings, it still retains its efficiency and superiority when compared to other pairing-based IBI schemes.

## 1. Introduction

Identification schemes, first proposed by Fiat and Shamir [1], are a cryptographic primitive that allows one party, called the prover, to verify himself to another party, the verifier, with the verifier learning nothing else other than the fact that the prover knows the prover's secret key as claimed. This primitive is a challenge-response one-way authentication mechanism that is frequently used in access control and is able to provide high security guarantees due to the zero-knowledge property of the protocol.

Traditional identification schemes, however, rely on a certificate issued by a certificate authority to explicitly certify that a user's public key rightfully belongs to him. To mitigate the problem of cryptosystems growing large and where certificate management becomes a major and costly issue, Shamir proposed the notion of identity-based cryptography, where certificates are no longer necessary and users can implicitly certify their public keys using their own identity-string [2].

However, identity-based cryptography only began to gain interest in 2001 when Boneh and Franklin proposed the first identity-based encryption scheme [3]. Three years later in 2004, IBI schemes were then formalized independently by Bellare et al. [4] and Kurosawa and Heng [5].

### 1.1. Related Work

Reference [4] presented a framework to construct IBI schemes from traditional public key identification schemes using a family of trapdoor sampleable relations. The authors also showed the relationship between security notions of standard identification schemes, public key signature schemes, IBI schemes, and identity-based signature schemes.

On the other hand, [5] showed that any digital signature with a zero-knowledge proof-of-knowledge protocol can be converted into an IBI scheme that is secure against impersonation under passive attacks. Reference [6], which is an extension of [5], showed several instantiations of the Kurosawa-Heng transformation, among which was the Schnorr-IBI scheme which is based on the transformation from the Schnorr digital signature scheme [7].

This passive-secure original scheme was fast and efficient and based on the weak discrete logarithm assumption similar to the signature scheme it was derived from. However, to improve the scheme to be secure against impersonation under active and concurrent attacks, a modified strong witness hiding protocol was required. This yielded an inefficient scheme where the protocol had to be repeated *k* = log⁡_2_ *q* times, where *q* is the size of the discrete logarithm group.

Tan et al. improved on this result in 2011, modifying the Schnorr-IBI scheme to provide active and concurrent security using only one iteration of the protocol [8]. However, since it was basing its security on the decisional Diffie-Hellman assumption, which is a stronger assumption than the discrete logarithm assumption, it thus resulted in a degradation of security guarantee.

### 1.2. Motivations and Contributions

While pairing-based IBI schemes continue to flourish, it would be of interest to continue to improve existing pairing-free IBI schemes in terms of both efficiency and security. It is well known that pairing operations are costly compared to other operations like exponentiations, as shown by implementation results such as those by [9]. Therefore, pairing-free IBI schemes run faster than their pairing-based counterparts in general.

In this paper, we show that the Schnorr-IBI scheme is able to be proven secure against impersonation under active and concurrent attacks using only the classical discrete logarithm assumption, which is an improvement in terms of security guarantee over the results of [8] of using the decisional Diffie-Hellman assumption. This comes at a small cost to storage and operation.

Specifically, we extend the number of secret key components to two components and thus name the modified Schnorr scheme the Twin-Schnorr-IBI scheme. The increased security guarantee comes at a small price in terms of increase in user secret key size as well as a few additional exponentiation operations. However, when compared to the majority of IBI schemes that utilize bilinear pairings, it is still considered more efficient since it is pairing-free.

One of the desirable properties of identification schemes is in their fast operation sequence. Generally speaking, three-move identification schemes are one of the faster cryptographic primitives in the asymmetric cryptography setting. This can be seen via the Fiat-Shamir transform, where digital signatures are constructed from identification schemes. This fast operation, coupled with the security feature where no information can be obtained by observing the running of the identification protocol, makes the identification scheme an excellent candidate for implementation on low-power, light-processing platforms. Moreover, for pairing-free identity-based identification schemes, one is able to authenticate himself securely with minimal computing required because certificate management issues are a thing of the past, and no pairing operations means less power is required in computing the intermediate steps during the identification protocol.

With strong security guarantees, high efficiency without pairing operations, and in the identity-based setting, the Twin-Schnorr-IBI scheme fits the description of the lightweight cryptographic scheme that is an excellent cryptographic primitive that can be implemented to provide fast access control mechanisms for entity authentication without having to use certificates. Implementation instances of the scheme can be applied to smart cards, mobile devices, and online systems to facilitate entity authentication before granting these entities access to available resources such as the larger reservoirs of computing power on cloud servers to handle the subsequent more taxing computations.

The rest of the paper is organized as follows. In Section 2, we begin with some preliminaries, including assumptions and security definitions for IBI schemes. In Section 3 we propose the Twin-Schnorr-IBI scheme. In Section 4 we provide a corresponding proof of security against impersonation under active and concurrent attacks. In Section 5 we provide a comparison of our proposed scheme against other discrete logarithm-based IBI schemes provable secure in the random oracle model. In Section 6, we provide implementation results to demonstrate the speed of the Twin-Schnorr-IBI scheme. We provide some areas of potential application for the Twin-Schnorr-IBI scheme in Section 6 and conclude in Section 7.

## 2. Preliminaries

### 2.1. Notations

Let {0,1} be the set of individual bits and let {0,1}^*^ be the set of all bit strings. Let *N* = {0,1, 2,…} denote the set of natural numbers, and if *k* ∈ *N* then 1^*k*^ is the bit string of *k* ones. Denote by {0,1}^*n*^ the set of bit strings of length *n* ∈ *N*. If *x* is a binary string, we denote by |*x*| its length and denote by *x*∣*y* the concatenation of strings *x* and *y*.

If *A* is a set then by $a\overset{\text{\$}}{\leftarrow }A$ we denote the action of sampling *a* uniformly from *A*. If *A* is an algorithm then *a* ← *A*(*x* : *r*) denotes that *A* outputs *a* when run with input *x* and random coins *r*. By *A*(*x*) we denote the distribution of *a* ← *A*(*x* : *r*) over the uniform choice of *r*. For algorithms *X* and *Y*, denote by *a* ← *X*(·)↔*Y*(·) an output *a* produced as the result of an interaction between *X* and *Y* on arbitrary inputs.

Also, define a negligible function *f* : *N* → [0,1] such that, for every positive exponent *c* ∈ *N*, there exists an integer *k*_*c*_ ∈ *N* such that *f*(*k*) ≤ *k* ^−*c*^ for all *k* > *k*_*c*_.

### 2.2. Discrete Logarithm Assumption

Let *G* be a cyclic group with prime order *q* and let *g* be a generator of *G*. The discrete logarithm problem is defined as follows: given a number *A* = *g* ^*a*^ in group *G*, output *a*.

The discrete logarithm assumption states that there exists no polynomial time algorithm *M* that is able to (*t*′, *ε*′)-solve the discrete logarithm problem with nonnegligible probability such that *Pr*⁡⁡[*M*(*G*, *q*, *g*, *A*)] ≥ *ε*′.

### 2.3. Formal Definition of IBI Schemes

An IBI scheme is defined by four polynomial time algorithms SETUP, EXTRACT, PROVE, and VERIFY.

1. SETUP takes in the security parameter (1^*k*^) and outputs the master public key *mpk* and master secret key *msk*.
2. EXTRACT takes in *mpk*, *msk*, and the user identity-string *ID* to produce the user secret key *usk*.
3. IDENTIFICATION PROTOCOL: PROVE and VERIFY interact with each other according to a three-step canonical proof-of-knowledge protocol. Each algorithm takes in *mpk* and *ID*, with PROVE receiving the additional *usk* as auxiliary input.
  - PROVE initiates and sends a commitment to VERIFY. Usually this takes the form of the prover's identity-string mixed with some salt for the identification instance.
  - VERIFY picks a random challenge from a set of predefined challenges to send to PROVE. These challenges are uniformly distributed within the predefined set of challenges.
  - PROVE responds with an answer to the challenge and VERIFY chooses whether to accept or reject based on PROVE's response that is calculated based on the identification instance's commitment, challenge, and the user secret key *usk* and identity-string *ID*.

The definition of an IBI scheme is presented in Algorithm 1.

### 2.4. Security Model for IBI Schemes

An adversary attacking an IBI scheme is defined as an impersonator. The goal of the impersonator is to successfully impersonate an honest user. Impersonators are further broken down into two categories.

- Passive impersonator: this type of impersonator only eavesdrops on conversations between honest users and verifiers before attempting impersonation.
- Active and concurrent impersonators: these impersonators are able to actively participate in conversations with honest verifiers to learn as much information as they can before attempting impersonation. The difference between active and concurrent impersonators is that active impersonators are only able to interact with one verifier at a time, while the concurrent impersonator is able to run several conversations with several verifiers concurrently.

We model the security of an IBI scheme as a game played by an impersonator *I* and a challenger *C* as follows.

1. *C* creates *mpk* and *msk* and runs *I* as a subroutine. *C* passes *mpk* to *I* but keeps *msk* to itself.
2. In phase 1, *I* can issue extract and identification queries. If *I* is a passive impersonator, *C* will respond with transcripts of valid conversations between honest provers and honest verifiers for an identification query. If *I* is an active and concurrent impersonator, *C* will play the cheating verifier to answer *I*'s identification queries while *I* plays the role of the prover.
3. In phase 2, *I* outputs an identity that it wishes to be challenged on. *I* is able to continue issuing extract and identification queries, but once it converses with *C* as a cheating prover and manages to convince *C* that he is the challenge identity, he wins the game.

We provide the description of the experiment in Algorithm 2.

Let *I* = (*CV*, *CP*) who runs in time *t* with *atk* ∈ {*pa*, *aa*, *ca*} and security parameter *k*; the advantage of *I* to impersonate is

(1) $$\begin{array}{c} Adv_{IBI,I}^{imp\text{-}atk}\left(k\right)=\mathrm{Pr}\left[{Exp}_{IBI,C,I}^{imp\text{-}atk}\left(k\right)=1\right], \end{array}$$

where *I* can make at most *q*_*e*_ extract queries. An IBI scheme is *imp*-*atk* secure if *Adv*_*IBI*,*I*_^*imp*-*atk*^(·) is negligible for every polynomial time *I*.

## 3. The Twin-Schnorr-IBI Scheme

Before the description of the scheme, we briefly note that while the key generation process of Schnorr-IBI from [5] looks similar to that of BNN-IBI from [4], the two schemes have different identification protocols and are therefore considered as two distinct IBI schemes. Similarly, one might note the similarity in the key generation process for OKDL-IBI from [4] with Twin-Schnorr-IBI, but because the two schemes have separate and distinct identification protocols, we consider them as two distinct IBI schemes.

The construction of the Twin-Schnorr-IBI scheme is as follows.

1. SETUP takes in the security parameter 1^*k*^ and generates the group *G* of order *q*. It picks random generators $g_{1},g_{2}\overset{\text{\$}}{\leftarrow }G$ and two random integers $x_{1},x_{2}\overset{\text{\$}}{\leftarrow }Z_{q}$. It sets *X* = *g*_1_^−*x*_1_^ *g*_2_^−*x*_2_^ and lastly chooses a hash function *H* : {0,1}^*^ × *G* × *G* → *Z*_*q*_. It publishes *mpk* = 〈*G*, *q*, *g*_1_, *g*_2_, *X*〉 while keeping *msk* = 〈*x*_1_, *x*_2_〉 securely stored away.
2. EXTRACT takes in *mpk* = 〈*G*, *q*, *g*_1_, *g*_2_, *X*〉, *msk* = 〈*x*_1_, *x*_2_〉, and the user identity-string *ID*. It first picks two random integers $r_{1},r_{2}\overset{\text{\$}}{\leftarrow }Z_{q}$ and calculates *α* = *H*(*ID*, *R*, *X*) and sets *R* = *g*_1_^*r*_1_^ *g*_2_^*r*_2_^. With those values, EXTRACT then calculates *s*_1_ = *r*_1_ + *x*_1_ *α* and *s*_2_ = *r*_2_ + *x*_2_ *α* and sets *usk* = 〈*s*_1_, *s*_2_, *α*〉.
3. IDENTIFICATION PROTOCOL: PROVE takes in *mpk*, *ID*, and *usk* while VERIFY takes in *mpk* and *ID*. They run the identification protocol as follows.
  - PROVE begins by picking two random integers $y_{1},y_{2}\overset{\text{\$}}{\leftarrow }Z_{q}$ and sets *Y* = *g*_1_^*y*_1_^ *g*_2_^*y*_2_^. PROVE additionally sets *R* = *g*_1_^*s*_1_^ *g*_2_^*s*_2_^ *X* ^*α*^ and sends *Y*, *R* to VERIFY.
  - VERIFY picks a random challenge $c\overset{\text{\$}}{\leftarrow }Z_{q}$ and sends it to PROVE.
  - PROVE responds by setting *z*_1_ = *y*_1_ + *cs*_1_ and *z*_2_ = *y*_2_ + *cs*_2_ and then sends *z*_1_, *z*_2_ to VERIFY as its response.

VERIFY calculates *α* = *H*(*ID*, *R*, *X*) and VERIFY accepts if the following equation holds:

(2) $$\begin{array}{c} g_{1}^{z_{1}}g_{2}^{z_{2}}=Y{\left(\frac{R}{X^{\alpha }}\right)}^{c}. \end{array}$$

VERIFY can calculate *α* = *H*(*ID*, *R*, *X*) by itself since

(3) g1s1g2s2Xα=g1r1+x1αg2r2+x2αg1−x1αg2−x2α=g1r1g2r2=R.

Correctness of the identification protocol can be proven as such:

(4) YRXαc=g1y1g2y2g1s1g2s2XαXαc=g1y1g2y2g1s1g2s2c=g1y1g2y2g1cs1g2cs2=g1y1+cs1g2y2+cs2=g1z1g2z2.

The detailed description of the scheme is provided in Algorithm 3.

## 4. Security Analysis

To outline the strategy for the proof, the general strategy is to prove that a challenger to the discrete logarithm problem can use the impersonator for the Twin-Schnorr-IBI scheme to solve an instance of the discrete logarithm problem.

The challenge takes in the discrete logarithm problem instance and simulates the environment for the Twin-Schnorr-IBI for the learning phase of phase 1.

In phase 2, it then uses the impersonation attempt of the Twin-Schnorr-IBI to solve the instance of the discrete logarithm problem. However, it is well known that no probabilistic polynomial time algorithm can solve the discrete logarithm problem.

Hence we arrive at the conclusion that no such impersonator for the Twin-Schnorr-IBI exists.

**Theorem 1 .**

The Twin-Schnorr-IBI scheme is secure against impersonation under active and concurrent attacks if the discrete logarithm problem is hard in group *G*, where

(5) $$\begin{array}{c} Adv_{IBI,I}^{imp\text{-}aa/ca}\leq \sqrt{Adv_{G,M}^{d\log}\left(k\right)+\frac{1}{2^{k}}}+\frac{1}{2^{k}}. \end{array}$$

**Proof**

Suppose an impersonator *I* exists. We can then show an algorithm *M* that is able to break the discrete logarithm problem. *M* begins by taking in the discrete logarithm instance (*g*, *A* = *g* ^*a*^) and runs *I* as a subroutine to calculate *a*.

*M* simulates the challenge environment for *I* by first setting *g*_1_ = *g* and *g*_2_ = *A*. Then it runs the rest of SETUP as usual, choosing two random integers $x_{1},x_{2}\overset{\text{\$}}{\leftarrow }Z_{q}$ and setting *X* = *g*_1_^−*x*_1_^ *g*_2_^−*x*_2_^, publishing *mpk* = 〈*G*, *q*, *g*_1_, *g*_2_, *X*〉 while keeping *msk* = 〈*x*_1_, *x*_2_〉 to itself.

In phase 1, *I* plays the cheating verifier in training, trying to learn what it can from *M*. As defined in the security model, *I* is able to issue both extract and identification queries.

Throughout the simulation, *M* instantiates a user with credentials via the *INIT*(*ID*_*i*_) oracle and adds to the set of generated identities *HU* and their corresponding *usk*. Also define the set *CU* to be the set of all corrupted users on which *I* has issued an extract query (and therefore corrupted). When *I* queries *usk*_*ID*_*i*__ for *ID*_*i*_ via the corrupt oracle *CORR*(*ID*_*i*_), *M* will generate *usk*_*ID*_*i*__ using the original EXTRACT algorithm since *M* has access to *msk*, removes *ID*_*i*_ from *HU*, and then adds *ID*_*i*_ to the set *CU*. Therefore, *q*_*e*_ = |*CU*| represents the maximum number of corrupt queries that *I* can issue.

Similarly, when *I* issues identification queries on *ID*_*j*_, *M* will create *usk*_*ID*_*j*__ (if not yet queried before as an extract query) to participate in the identification protocol oracle *IDENT*(*ID*_*i*_) as a prover and adds 〈*ID*_*j*_, *usk*_*ID*_*j*__〉 to the set *HU*. Without loss of generality we can assume that *I* will not issue an identification query on a user which it has corrupted already. It is evident that *I* can query as many identification queries as it likes as long as the number of *usk* generated does not exceed *q*_*e*_.

In phase 2, *I* takes on the role of the cheating prover trying to convince *M* that it knows *usk*_ID^*^_ of the challenge identity *ID* ^*^. *I* outputs *ID* ^*^ and then runs the identification protocol oracle *IDENT*(*ID* ^*^) with *M*, but this time as a cheating verifier. If *M* does not have *usk*_ID^*^_ = 〈*s*_1_, *s*_2_〉 generated yet prior to this, it runs *INIT*(*ID*_*i*_) to create it now.

Using the Reset Lemma [10], *M* is able to extract 2 valid transcripts $\left[\tilde{Y},\tilde{R},\tilde{c_{1}},\tilde{z_{1,1}},\tilde{z_{1,2}}\right]$ and $\left[\tilde{Y},\tilde{R},\tilde{c_{2}},\tilde{z_{2,1}},\tilde{z_{2,2}}\right]$ from *I* where $\tilde{c_{1}}\neq \tilde{c_{2}}$. From here, *M* extracts $\tilde{s_{1}}=\left(\tilde{z_{2,1}}-\tilde{z_{1,1}}\right)/\left(\tilde{c_{2}}-\tilde{c_{1}}\right)$ and $\tilde{s_{2}}=\left(\tilde{z_{2,2}}-\tilde{z_{1,2}}\right)/\left(\tilde{c_{2}}-\tilde{c_{1}}\right)$. If $s_{1}=\tilde{s_{1}}$ and $s_{2}=\tilde{s_{2}}$ then *M* aborts. The attack on the discrete logarithm problem has failed. Otherwise, if the values are different, *M* can calculate *a* as

(6) $$\begin{array}{c} g_{1}^{\tilde{s_{1}}}g_{2}^{\tilde{s_{2}}}=g_{1}^{s_{1}}g_{2}^{s_{2}},\\ g^{\tilde{s_{1}}+a\tilde{s_{2}}}=g^{s_{1}+as_{2}},\\ g^{a\tilde{s_{2}}-as_{2}}=g^{s_{1}-\tilde{s_{1}}},\\ g^{a}=g^{\left(s_{1}-\tilde{s_{1}}\right)/\left(\tilde{s_{2}}-s_{2}\right)},\\ a=-\left(\frac{\tilde{s_{1}}-s_{1}}{\tilde{s_{2}}-s_{2}}\right). \end{array}$$

A detailed description of the Simulator *M* that solves the discrete logarithm problem using impersonator *I* as subroutine is given in Algorithm 4.

It remains to calculate the probability of *M* winning the game to solve the discrete logarithm problem. By the Reset Lemma, *M* will successfully extract 2 valid conversations to derive (*s*_1_, *s*_2_) and calculate *a* with probability (*Adv*_*IBI*,*I*_^*imp*-*aa*/*ca*^ − 1/2^*k*^)^2^.

Let the advantage of *M* solving the discrete logarithm problem be defined as *Adv*_*G*,*M*_^*d*log⁡^(*k*), which comprises the events *M* computes *a*, defined as event *A*, and that *M* does not abort, defined as event *B*. The probability that *M* wins is given by

(7) AdvG,Mdlog⁡k=Pr⁡⁡A∧B=Pr⁡⁡A ∣ BPr⁡⁡B,AdvG,Mdlog⁡k≥ε−12k2Pr⁡⁡B.

The probability of *M* aborting, event *B*, is exactly 1/2^*k*^ since the only event that *M* aborts is if $s_{1}=\tilde{s_{1}}$ and $s_{2}=\tilde{s_{2}}$.

Hence the probability of *M* winning is

(8) $$\begin{array}{c} Adv_{G,M}^{d\log}\left(k\right)\geq {\left(Adv_{IBI,I}^{imp\text{-}aa/ca}-\frac{1}{2^{k}}\right)}^{2}-\frac{1}{2^{k}},\\ Adv_{G,M}^{d\log}\left(k\right)+\frac{1}{2^{k}}\geq {\left(Adv_{IBI,I}^{imp\text{-}aa/ca}-\frac{1}{2^{k}}\right)}^{2},\\ Adv_{IBI,I}^{imp\text{-}aa/ca}\leq \sqrt{Adv_{G,M}^{d\log}\left(k\right)+\frac{1}{2^{k}}}+\frac{1}{2^{k}}. \end{array}$$

## 5. Efficiency Analysis

In this section, we provide the efficiency cost of the Twin-Schnorr-IBI scheme in Table 1. We measure the operation costs in terms of exponentiations, multiplications in group *G*, multiplications in *Z*_*q*_, and additions in *Z*_*q*_.

Next a comparison is made with other IBI schemes that are constructed based on discrete logarithms in Table 2. We take into consideration only the identification protocol operation cost, since that is the most-run algorithm in the whole scheme.

From the comparisons above in Table 2, one can see that the closest competitor for Twin-Schnorr is the OKDL-IBI scheme since both offer almost similar efficiency cost using the classical discrete logarithm assumption. In fact, for the schemes as they are, OKDL-IBI is slightly more efficient with one less exponentiation and one less multiplication in *G* in its identification protocol.

However, it is possible to increase the efficiency of both Twin-Schnorr-IBI and OKDL-IBI through precomputation of some of the fixed values in the identification protocol. For example, for Twin-Schnorr-IBI, *V* can be computed beforehand and stored in the PROVE party's device for future use since the value does not change. For OKDL-IBI, one can first precompute and store *S* = *g*_1_^−*s*_1_^ *g*_2_^−*s*_2_^ for similar reasons, resulting in less exponentiations. Results of the improved efficiency of the Twin-Schnorr-IBI and OKDL-IBI after precomputation are presented in Table 3.

If precomputation is done, then it is shown that the two schemes have similar operation costs. However, Twin-Schnorr requires less communication bandwidth of 1 group element in *G* (generally 2048 bits for 128-bit security) compared to OKDL-IBI for each run of the identification protocol.

Secondly, Twin-Schnorr-IBI does not require the semistrong unforgeability property, as defined by [4], since no component of the user secret key is transmitted in the open. This is in contrast to the OKDL-IBI scheme, where one component of the user secret key is transmitted from PROVE to VERIFY during each interaction. Thus, there is security degradation with the requirement of semistrong unforgeability for the OKDL-IBI.

Therefore, with precomputation, the Twin-Schnorr-IBI is slightly superior in terms of efficiency and security compared to the OKDL-IBI.

## 6. Implementation Results

In this section, we run an instantiation of the Twin-Schnorr-IBI written in Java utilizing the Java Cryptography Architecture and Java Cryptography Extension libraries to showcase the actual running time results. The simulation was conducted on an i7-4702 MQ workstation with 8 GB RAM running on 64-bit Windows 8 operating system. Each algorithm of the Twin-Schnorr-IBI scheme is run for 100 times using various security-level settings and the average running time is taken. The results are displayed in Table 4.

## 7. Potential Applications of Twin-Schnorr-IBI

As mentioned in the introduction, the Twin-Schnorr-IBI scheme is ideal in terms of providing a lightweight secure-authentication mechanism to facilitate access control to further resources. One desirable area of facilitating access control would be in multimedia systems, where the Twin-Schnorr-IBI can be used to determine which user is allowed access to certain multimedia content. This is implementable on multimedia services mentioned in [11], IPTV service platforms [12], and even learning management systems [13]. Another area where secure identification is required is to determine user access to confidential data. Access to job information systems [14], credit-card payment systems [15], and ID credit scoring systems [16] would be suitable for the Twin-Schnorr-IBI to be implemented as a gateway before allowing users access to the confidential information found within the systems. The Twin-Schnorr-IBI scheme can also be used for peer-to-peer validation before allowing users into a hosted e-meeting such as ones conducted using the shared presentation board [17]. Another application for the Twin-Schnorr-IBI is to serve as an initial identification before allowing users further access to cloud-based computing resources such as cloud-based personal tutoring systems [18] and personal mobile albums and diaries [19]. As one can see, the use of the IBI scheme can be versatile for use in multiple settings, and the Twin-Schnorr is a good instantiation to serve these purposes because of its fast running time.

## 8. Conclusion

In this paper, we showed that, with a slight additional cost to the original Schnorr-IBI scheme, we can obtain security against active and concurrent impersonation attacks using the weak discrete logarithm assumption and thus provide a high security guarantee compared to the single key counterpart that rely on the stronger decisional Diffie-Hellman assumption. The modified scheme is efficient in that it remains pairing-free and is comparable (and slightly more secure and efficient) to the most-secure pairing-free IBI scheme in literature to date in terms of efficiency under the classical discrete logarithm assumption.

## Figures and Tables

**Algorithm 1:**
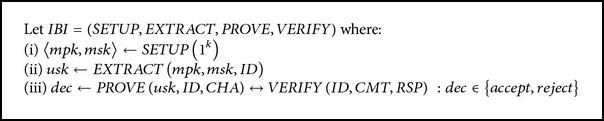
Definition of an IBI scheme.

**Algorithm 2:**
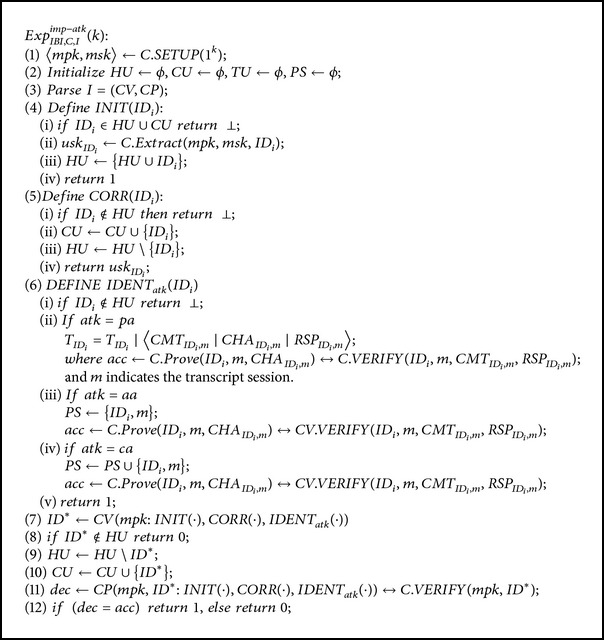
Description of the security model for IBI schemes.

**Algorithm 3:**
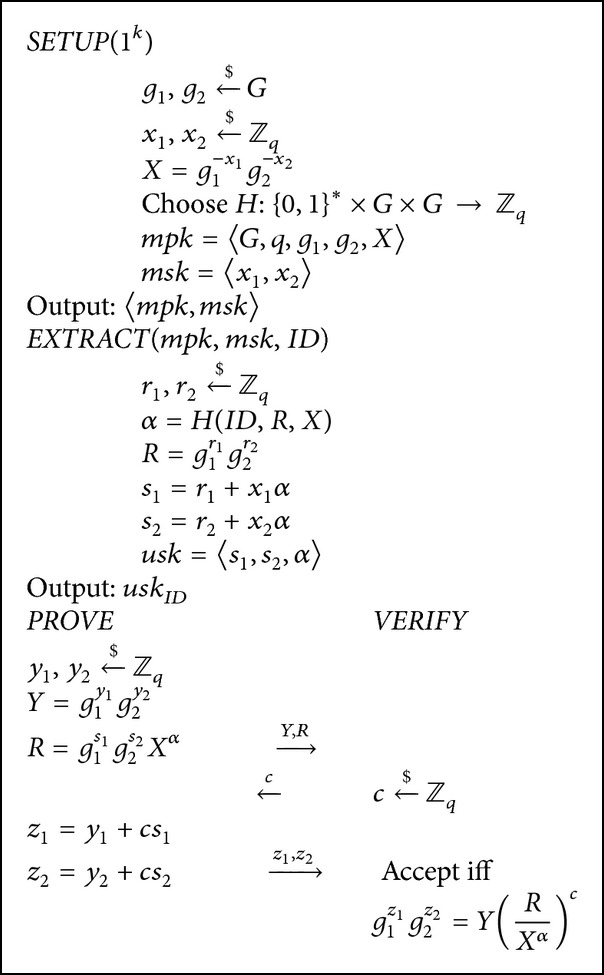
Definition of the Twin-Schnorr-IBI scheme.

**Algorithm 4:**
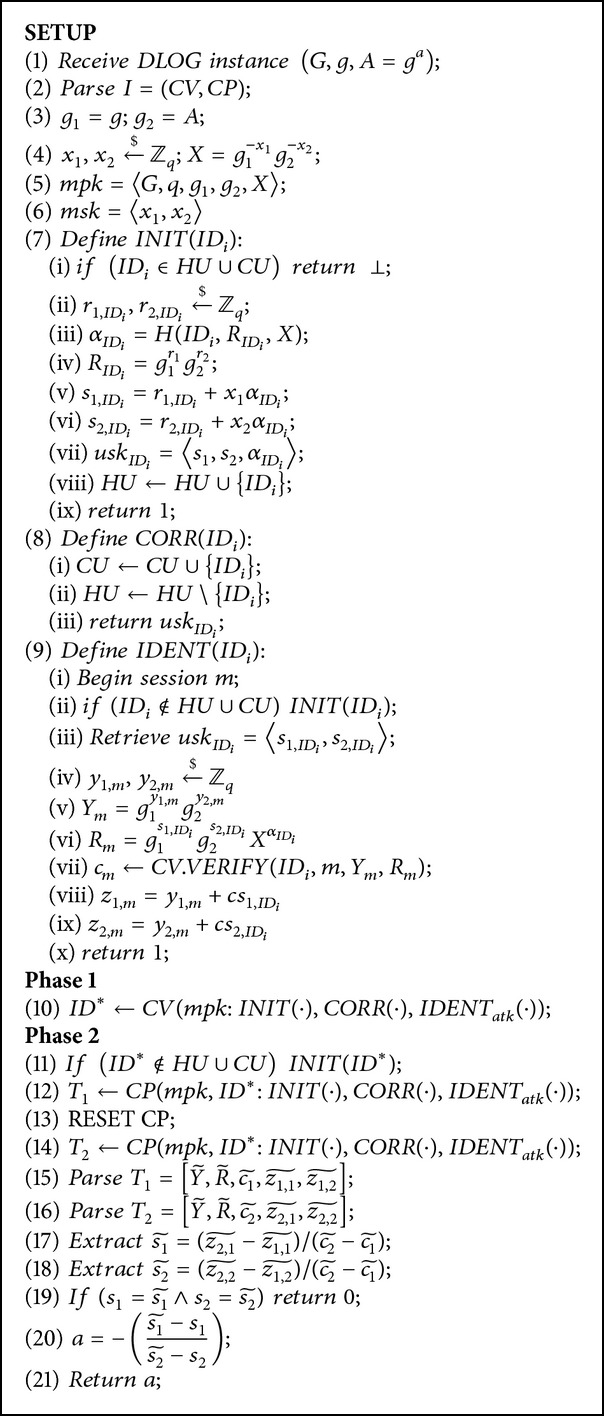
Description of the Simulator *M* solving an instance of the discrete logarithm problem with the help of impersonator *I* as subroutine.

**Table 1:** Efficiency of the Twin-Schnorr-IBI Scheme without precomputation.

Algorithm	E	MG	MZ	A
SETUP	2	1	0	0
EXTRACT	2	1	2	2
PROVE	5	3	2	2
VERIFY	4	3	0	0

E: exponentiation, MG: multiplication in *G*, MZ: multiplication in *Z*_*q*_, and A: addition in *Z*_*q*_.

**Table 2:** Comparison of the identification protocol with other discrete logarithm IBI schemes.

Scheme	U	E	MG	MZ	IMP-AA/CA
OKDL-IBI (Bellare et al., 2004 [4])	3	8	5	2	DLP
BNN-IBI (Bellare et al., 2004 [4])	2	5	2	1	OMDLP
Beth-IBI (Bellare et al., 2004 [4])	2	4	2	3	Unknown
Schnorr-IBI (Heng, 2004 [6])	2	6*k*	3*k*	*k* ^*^	DLP
Tight-Schnorr-IBI (Tan et al., 2011 [8])	2	6	3	1	DDHP
Twin-Schnorr-IBI	3	9	6	2	DLP

U: *usk* components, E: exponentiation, MG: multiplication in *G*, MZ: multiplication in *Z*_*q*_, IMP-AA/CA: security assumption against impersonation under active and concurrent attack, DLP: discrete logarithm problem, OMDL: one-more discrete logarithm problem, and DDHP: decisional Diffie-Hellman problem, *k* = log_2_⁡*q*.

**Table 3:** Comparison of the identification protocol of Twin-Schnorr-IBI and OKDL-IBI after precomputation.

Scheme	E	MG	MZ	Communication costs
OKDL-IBI	6	4	2	3*G* + 3*Z*
Twin-Schnorr-IBI	6	4	2	2*G* + 3*Z*

E: exponentiation, MG: multiplication in *G*, MZ: multiplication in *Z*_*q*_, *G*: element in *G*, and *Z*: element in *Z*_*q*_.

**Table 4:** Average running time of Twin-Schnorr-IBI algorithms on different security levels.

	Setup (ns)	Extract (ns)	Identification protocol (ns)
1024 bits	4,418,212,851	769,496	4,061,664
2048 bits	71,367,733,000	3,583,750	19,404,604
3072 bits	161,592,868,889	7,506,387	43,790,964
